# P15 JAKi of all trades

**DOI:** 10.1093/rap/rkad070.036

**Published:** 2023-09-27

**Authors:** Clara Watkins, Deepak Nagra, Edward Alveyn, Maryam Adas, Chris Baldwin, James Galloway

**Affiliations:** Department of Rheumatology, London, United Kingdom; Department of Rheumatology, London, United Kingdom; Department of Clinical and Academic Rheumatology, London, United Kingdom; Department of Rheumatology, London, United Kingdom; Department of Clinical and Academic Rheumatology, London, United Kingdom; Department of Rheumatology, London, United Kingdom; Department of Clinical and Academic Rheumatology, London, United Kingdom; Department of Rheumatology, London, United Kingdom; Department of Rheumatology, London, United Kingdom; Department of Clinical and Academic Rheumatology, London, United Kingdom

## Abstract

**Introduction:**

Sarcoidosis affects approximately 1% of the population. Of these, up to one-third of patients will develop cutaneous manifestations. There are two situations where sarcoidosis can become life threatening; seizures secondary to neuro-sarcoidosis and arrhythmias due to cardiac infiltration. Currently, there are limited licensed therapies for the treatment of sarcoidosis. We describe a case of multi-system sarcoidosis whose extensive skin and pulmonary manifestations did not respond to conventional DMARDs and biologics requiring targeted therapies, in the context of recent military service.

**Case description:**

A 46-year-old male who worked as a Corporal in the Army presented with shortness of breath, blurry vision and painful red eyes to his local hospital. His symptoms had been ongoing for a few weeks prior to presentation. On examination he had reduced air entry in his lungs. His heart sounded regular; however, it was noted that he had erythema nodosum on his shins alongside tender nodular skin lesions on his elbows, feet and arms. He had synovitis in his MCPs and PIPs bilaterally. A chest X-ray demonstrated bilateral hilar lymphadenopathy and routine blood work was unremarkable. He was referred to the ophthalmologists who noted he had bilateral panuveitis with retinal vasculitis. His QuantiFERON, HIV and syphilis serology were negative; however, his hepatitis B core antibody was positive with a negative DNA. A skin biopsy confirmed non-necrotising granulomas and a subsequent HRCT demonstrated small volume bilateral pleural effusions, central mediastinal and hilar lymphadenopathy with micronodular changes. An EBUS confirmed non-necrotising, non-caseating epithelioid granulomas consistent with a diagnosis of sarcoidosis. He was commenced on a tapering dose of prednisolone (30mg to start with), hydroxychloroquine (400mg) and methotrexate (15mg). He remained well until the return of his lesions 24 months later. On assessment, his skin lesions had returned with dactylitis in his toes and synovitis in his wrists. Investigations revealed no improvement in his vital capacity and a markedly raised Il2Ra of 5739ng/L which was previously 2812ng/L (reference range 423-1843ng/L). A decision was made to commence infliximab (5mg/kg) to manage his inflammatory arthritis and cutaneous sarcoidosis. He remained well on infliximab for 6 months with the recurrence of his skin lesions requiring repeated courses of prednisolone (30mg). A CT-PET demonstrated active pulmonary and skin lesions. A decision to escalate his treatment was made.

**Discussion:**

Our patient had both biochemical and radiological evidence of cutaneous and pulmonary sarcoidosis affecting his quality of life both physically and psychologically. He has significant exertional breathlessness, skin lesions across his torso and abdomen, and scored highly for depression and anxiety on GAD7 and PHQ9 respectively. There are few therapies available for the management of sarcoidosis beyond TNF inhibitors; however, there are promising clinical trials for the use of tofacitinib in cutaneous sarcoidosis. Our patient demonstrated both a seronegative inflammatory arthritis alongside his sarcoidosis and a decision was made to commence tofacitinib 5mg twice daily. His baseline DAS-28 score was 5.81. His skin lesions have gradually resolved 8 weeks after starting treatment and he has had an improvement in his respiratory and musculoskeletal symptoms. This is a very promising initial result for a treatment currently undergoing clinical trial for use in sarcoidosis.

It is likely that his occupational risk factors could have triggered the sarcoidosis initially. He worked as a Corporal in the Army including 3 tours to Iraq over a 5-year period and would have been exposed to unspecified particular matter. Presentations of sarcoidosis following military service have been described previously. Similarly, there was a large spike in incidence of pulmonary sarcoidosis reported following the collapse of the Twin Towers. This is thought to be related to the increase in aerosol particular matter following the disaster, triggering an immune response when inhaled in susceptible adults. This has the potential to have financial implications for the patient.

**Key learning points:**

Sarcoidosis remains a challenging disease to diagnose and manage given its heterogenicity, but has potentially fatal consequences if left untreated. There is no single diagnostic test. Presentation varies from innocent bystander with isolated lymph node disease, to developing high disease burden with systemic involvement. This case highlights the potential for environmental factors to trigger sarcoidosis in susceptible patients. JAK inhibitors are the latest development in the field of sarcoidosis. Currently the only recognised JAK inhibitor for the treatment of sarcoidosis is tofacitinib as it inhibits both interferon by JAK1 and G-CSF through JAK2/3. STAT1 has been isolated during immunohistological analysis of skin lesions in sarcoidosis which is inhibited by tofacitinib and forms the basis of its use in cutaneous sarcoidosis. The management of sarcoidosis in the acute setting is commonly prednisolone; however, steroid sparing therapies should be considered in patients needing recurrent, prolonged courses of corticosteroids or those with evidence of critical organ involvement such as CNS, cardiac or lung disease. Life threatening disease or disease refractory to DMARD therapy should be considered for anti-TNF therapy with infliximab or adalimumab. More recently, tofacitinib has been promising as a treatment for sarcoidosis as seen in this case with clinical trials underway in Yale and Portland. Newer therapies in development include the GM-CSF inhibitor namilumab, and efzofitimod, a neuropilin 2 inhibitor.

